# Loss of p53 function promotes DNA damage-induced formation of nuclear actin filaments

**DOI:** 10.1038/s41419-023-06310-0

**Published:** 2023-11-25

**Authors:** Takeru Torii, Wataru Sugimoto, Katsuhiko Itoh, Natsuki Kinoshita, Masaya Gessho, Toshiyuki Goto, Ikuno Uehara, Wataru Nakajima, Yemima Budirahardja, Daisuke Miyoshi, Takahito Nishikata, Nobuyuki Tanaka, Hiroaki Hirata, Keiko Kawauchi

**Affiliations:** 1 Faculty of Frontiers of Innovative Research in Science and Technology (FIRST), Konan University, Kobe, 650-0047 Japan; 2 Department of Molecular Oncology, Institute for Advanced Medical Sciences, Nippon Medical School, Tokyo, 113-8602 Japan; 3 Department of Applied Bioscience, Kanazawa Institute of Technology, Hakusan, 924-0838 Japan

**Keywords:** Nucleoskeleton, Tumour-suppressor proteins

## Abstract

Tumor suppressor p53 plays a central role in response to DNA damage. DNA-damaging agents modulate nuclear actin dynamics, influencing cell behaviors; however, whether p53 affects the formation of nuclear actin filaments remains unclear. In this study, we found that p53 depletion promoted the formation of nuclear actin filaments in response to DNA-damaging agents, such as doxorubicin (DOXO) and etoposide (VP16). Even though the genetic probes used for the detection of nuclear actin filaments exerted a promotive effect on actin polymerization, the detected formation of nuclear actin filaments was highly dependent on both p53 depletion and DNA damage. Whilst active p53 is known to promote caspase-1 expression, the overexpression of caspase-1 reduced DNA damage-induced formation of nuclear actin filaments in p53-depleted cells. In contrast, co-treatment with DOXO and the pan-caspase inhibitor Q-VD-OPh or the caspase-1 inhibitor Z-YVAD-FMK induced the formation of nuclear actin filament formation even in cells bearing wild-type *p53*. These results suggest that the p53-caspase-1 axis suppresses DNA damage-induced formation of nuclear actin filaments. In addition, we found that the expression of nLifeact-GFP, the filamentous-actin-binding peptide Lifeact fused with the nuclear localization signal (NLS) and GFP, modulated the structure of nuclear actin filaments to be phalloidin-stainable in p53-depleted cells treated with the DNA-damaging agent, altering the chromatin structure and reducing the transcriptional activity. The level of phosphorylated H2AX (γH2AX), a marker of DNA damage, in these cells also reduced upon nLifeact-GFP expression, whilst details of the functional relationship between the formation of nLifeact-GFP-decorated nuclear actin filaments and DNA repair remained to be elucidated. Considering that the loss of p53 is associated with cancer progression, the results of this study raise a possibility that the artificial reinforcement of nuclear actin filaments by nLifeact-GFP may enhance the cytotoxic effect of DNA-damaging agents in aggressive cancer cells through a reduction in gene transcription.

## Introduction

Actin plays a crucial role in various cellular behaviors, not only cell morphogenesis and migration but also cell survival, proliferation, and differentiation, by regulating signal transduction [1–3]. In the steady state, filamentous actin (F-actin) continuously assembles and disassembles, and the amounts of monomeric globular actin (G-actin) and F-actin are maintained at equilibrium [4]. When cells are exposed to stressors, including DNA-damaging stimuli, this equilibrium is often disturbed, resulting in altered signal transduction. G-actin constantly shuttles between the cytoplasm and nucleus, which is regulated by importin-9 and exportin-6 [5, 6]. Many types of actin regulatory proteins, such as actin nucleators and actin filament-severing proteins, also translocate to the nucleus, resulting in the regulation of nuclear actin filament assembly [7, 8]. Nuclear F-actin regulates gene expression, chromatin architecture, nuclear envelope breakdown, and chromosome segregation [9–17]. Nuclear actin is usually difficult to detect, particularly in somatic cells; new probes for visualizing nuclear F-actin, including fluorescence-labeled actin and actin-binding proteins, such as actin chromobody and Lifeact, which are tagged with NLS, have been developed [18]. Using these probes, studies have revealed that DNA damage stimuli induce the formation of nuclear actin filaments, resulting in the promotion of DNA repair through the modulation of chromatin dynamics [8, 17].

The cellular response to DNA-damaging stimuli is critical for determining cell death. In the nuclei of cells with DNA damage, histone H2AX is phosphorylated in the chromatin region containing damaged DNA [19, 20]. Phosphorylated H2AX (γH2AX) acts as a docking site for DNA damage response/repair proteins, which promotes the efficient recruitment of these proteins to DNA damage sites and ensures the maintenance of the normal cell cycle by repairing damaged DNA [20–24]. In contrast, when the DNA damage is severe and irreparable, the apoptosis induction pathway is activated through the expression of several pro-apoptotic proteins, leading to the activation of caspases [25, 26]. Caspases, a family of cysteine proteases, are involved not only in apoptosis but also in inflammation [27]. During the process of apoptosis, effector caspases (caspase-3, caspase-6, and caspase-7), which execute apoptosis through the cleavage of target proteins, are activated by the initiator caspases (caspase-2, caspase-8, caspase-9, and caspase-10) [26]. On the other hand, inflammatory caspases (caspase-1, caspase-4, caspase-5, caspase-11, and caspase-12) induce the production of inflammatory mediators to generate active cytokines and often drive pyroptosis, another type of cell death [27, 28].

p53 (aka *TP53*) is the ‘guardian of the genome’ that responds to cellular stresses including DNA-damaging stimuli and protects the organism from cancer development and progression [29]. *TP53* is mutated in more than 50% of human cancers [30]. Normally, the activity of p53 is maintained at a low level [31, 32]. When cells are subjected to DNA-damaging stimuli, the activity of p53 increases. Activated p53 induces the expression of various target genes and exerts physiological effects, such as cell cycle arrest, DNA repair, and induction of apoptosis. The judgment of severe DNA damage as irreparable is associated with the phosphorylation of p53 at Ser46, which induces the expression of its target genes encoding pro-apoptotic proteins, leading to cell apoptosis through the activation of caspases [33]. Prolonged treatment with anticancer drugs sometimes makes p53 activation inefficient, causing cancer cell recurrence. Numerous studies have revealed that p53 suppresses the invasion and metastasis of cancer cells through the modulation of actin cytoskeletal structures under non-specific stress conditions, while only a few studies have investigated the mechanism of the p53-mediated regulation of the actin cytoskeleton [34–36]. However, whether p53 is involved in the formation of nuclear actin filaments in response to DNA-damaging agents remains unclear.

Here, we show that the DNA damage-induced formation of nuclear actin filaments is suppressed by p53. Caspases are involved in the p53-dependent suppression of nuclear actin filament formation. Furthermore, in p53-depleted cells expressing nLifeact-GFP, the DNA damage-induced formation of nuclear actin filaments was associated with a change in chromatin structure and a reduction in transcription. Whilst the loss of p53 is frequently observed in aggressive cancer cells, cancer progression might be reduced by modulating the structure of nuclear actin filaments through the introduction of nLifeact.

## Materials and methods

### Cell culture

MCF-7 human breast cancer cells, A549 human lung cancer cells, and 293T human embryonic kidney cells are obtained from the American Cells Type Culture Collection. Cells were cultured in Dulbecco’s modified Eagle’s medium (Nissui Pharmaceutical Co., Ltd., Tokyo, Japan) supplemented with 10% fetal bovine serum and 1% penicillin/streptomycin for MCF-7 cells, A549 cells, and 293T cells, or 50 μg/mL kanamycin for mouse embryonic fibroblasts (MEFs). MCF-7 cells expressing control shRNA or *p53* shRNA, *p53*^*+/+*^MEFs, and *p53*^*–/–*^MEFs were prepared as described previously [37, 38]. All animal experiments were approved by Konan University and conformed to the Guidelines for the Care and Use of Laboratory Animals published by the U.S. National Institutes of Health.

### Plasmids and transfection

The expression vectors nAC-GFP (nuclear Actin-Chromobody® plasmid) obtained from ChromoTek (Planegg, Germany), nLifeact-GFP (pEGFP-C1Lifeact 2XNLS, #58467) and YFP-nβ-actin WT or S14C (YFP NLS Beta-Actin WT or S14C, #60613 or #60614, respectively) obtained from Addgene (Watertown, MA, USA), and GFP (pEGFP-C1) obtained from Clontech Laboratories, Inc. (Mountain View, CA, USA) were used in this study. The *TP53* gene in pBabe p53 puro retroviral vector [38] was introduced as a silent mutation to inhibit the effect of its shRNA from [gactccagtggtaat] to [gattcgagcggaaac]. The mutant of *TP53* was amplified by PCR and then replaced with the *DMPK* gene in the pcDNA HA DMPK expression vector [36]. DNA encoding caspase-1 was obtained from a cDNA pool of A549 cells and replaced with the *HtrA2/Omi* gene in the pCMV-Flag HtrA2/Omi expression vector [39]. The transfection reagent PEI-MAX (Polysciences Inc., Warrington, PA, USA) was used for all cell types.

### Antibodies and materials

Anti-HA mouse monoclonal (16B12; Covance, Princeton, NJ, USA) and anti-γH2AX rabbit monoclonal (20E3; Cell Signaling Technology, Danvers, MA, USA) were used for immunofluorescence analysis and immunoblot analysis. Anti-histone H3 (acetyl K9) rabbit polyclonal (ab10812; abcam, Cambridge, UK) antibody was used for immunofluorescence analysis and immunoblot analysis. Anti-p53 mouse monoclonal (DO-1; Santa Cruz Biotechnology, Dallas, TX, USA, or 1C12; Cell Signaling Technology, Danvers, MA, USA), anti-Flag mouse monoclonal (M2; Sigma-Aldrich, St. Louis, MO, USA), anti-caspase 1 mouse monoclonal (14F468; Santa Cruz Biotechnology, Dallas, TX, USA), anti-γH2AX rabbit polyclonal (2577; Cell Signaling Technology, Danvers, MA, USA), anti-H2AX rabbit polyclonal (GeneTex, Inc., Irvine, CA, USA), and anti-α-tubulin mouse monoclonal (DM1A; Santa Cruz Biotechnology, Dallas, TX, USA) antibodies were used for immunoblot analysis. Doxorubicin (DOXO) and etoposide (VP16) were purchased from Calbiochem (La Jolla, CA, USA). Mycalolide B and cisplatin (CPT) were purchased from FUJIFILM (Tokyo, Japan). Q-VD-OPh was purchased from Sigma-Aldrich (St. Louis, MO, USA). Z-YVAD-FMK was purchased from abcam (Cambridge, UK).

### Immunofluorescence

Cells were fixed with 4% paraformaldehyde (PFA), permeabilized with 0.1% Triton X-100, and blocked with 2% BSA in phosphate-buffered saline (PBS). The cells were incubated with primary antibodies and subsequently with Alexa Fluor 647-conjugated goat anti-mouse IgG (Molecular Probes, Carlsbad, CA, USA) or Alexa Fluor 546-conjugated goat anti-rabbit IgG (Molecular Probes, Carlsbad, CA, USA) as the secondary antibody. Alexa Fluor 633 Phalloidin and DAPI (Vector Laboratories, Inc., Burlingame, CA, USA) were used to stain actin filaments and nuclei, respectively. Images were acquired using a confocal microscope (A1R HD25, Nikon Co., Tokyo, Japan) equipped with a water immersion objective lens (NA = 1.2, Plan Apo, Nikon Co., Tokyo, Japan), and were analyzed using ImageJ software [National Institute of Health (NIH), Bethesda, MD, USA]. The acquired γH2AX image was deconvoluted using the NIS Elements AR software, version 5.11.01.

### Analysis of nuclear actin filament formation

The cells expressing nAC-GFP were imaged using the confocal microscope A1R with a 60× objective lens (Nikon Co., Tokyo, Japan). The obtained images were analyzed using ImageJ software (NIH, Bethesda, MD, USA). First, images were segmented for individual cells using a saturated threshold established against nAC-GFP. Next, the background was subtracted using a 1-pixel radius rolling ball, and the proportion of nuclear area occupied by the nuclear actin filaments was subsequently measured. In cells without nuclear actin filaments, nAC-GFP was uniformly distributed inside the nucleus, whereas in the cells with nuclear actin filaments, nAC-GFP was mostly localized in the filaments, creating non-uniform nuclear localization. In most cells, nuclear actin filaments could be seen even when the distribution of nAC-GFP was only slightly non-uniform. Therefore, an area proportion of 1% was taken as the threshold to determine the presence of nuclear actin filaments. Cells with non-uniform nAC-GFP localization in ≥1% of their area were classified as nuclear actin filament-containing cells, whereas those in <1% were classified as nuclear actin filament-free cells.

### Immunoblot analysis

Immunoblot analysis was performed as described previously [37]. Cells were solubilized in lysis buffer (50 mM Tris pH 7.4, 150 mM NaCl, 1% Triton X-100, 1% SDS, 10 mM EDTA, 1 mM Na_3_VO_4_, 10 mM NaF, and protease inhibitor cocktail [Nacalai Tesque, Inc., Kyoto, Japan]) and then centrifuged at 20,000 × *g* for 15 min after sonication. The supernatants were used as total cell extracts and subjected to sodium dodecyl sulfate-polyacrylamide gel electrophoresis.

### Quantitative real-time PCR

Total RNA was isolated and purified using the NucleoSpin RNA kit (Takara Bio Inc., Shiga, Japan). For quantitative real-time PCR (qRT-PCR), cDNA was prepared using the PrimeScript 1st strand cDNA Synthesis kit (Takara Bio Inc., Shiga, Japan). qRT-PCR analysis was conducted with the cDNA of MCF-7 cells using THUNDERBIRD Next SYBR qPCR Mix (Toyobo, Osaka, Japan) under the following conditions: 30 s at 95 °C followed by 45 cycles at 95 °C for 5 s and 10 s at 60 °C using the StepOne Plus Real-Time PCR system (Applied Biosystems, Waltham, MA, USA). qRT-PCR analysis was conducted with cDNA of A549 cells using THUNDERBIRD SYBR qPCR Mix (Toyobo, Osaka, Japan) under the following conditions: 1 min at 95 °C, followed by 40 cycles at 95 °C for 15 s and 1 min at 55 °C using the StepOne Plus Real-Time PCR system. The following primers were used: human *CASP1* forward 5´-GCTGAGGTTGACATCACAGGCA-3´; human *CASP1* reverse 5´-TGCTGTCAGAGGTCTTGTGCTC-3´; human *UBC* forward 5´-TGACTACAACATCCAGAA-3´; human *UBC* reverse 5´-ATCTTTGCCTTGACATTC-3´. After normalization against human *UBC* mRNA, the relative expression levels to control were shown.

### Quantitative phase imaging and fluorescence imaging

Quantitative phase imaging images and the correlative fluorescence images of live cells were obtained using commercial holotomography (HT-2H, Tomocube Inc., Daejeon, South Korea), which was based on Mach-Zehnder interferometry and was equipped with a digital micromirror device (DMD). A coherent monochromatic laser (*λ* = 532 nm) was divided into two paths; a reference and a sample beam, respectively, using a 2 × 2 single-mode fiber coupler. A 3-D RI tomogram was reconstructed from multiple 2-D holographic images acquired from 49 illumination conditions, a normal incidence, and 48 azimuthally symmetric directions with a polar angle (64.5°). The DMD was used to control the angle of an illumination beam impinging onto the sample [40]. The diffracted beams from the sample were collected using a high numerical aperture (NA) objective lens (NA = 1.2, UPLSAPO 60XW, Olympus, Tokyo, Japan). The off-axis hologram was recorded by a CMOS image sensor (FL3-U3-13Y3MC, FLIR Systems, Wilsonville, OR, USA). For epifluorescence imaging, nAC-GFP and nLifeact-GFP were excited by an LED light source (470 nm). Deconvolution of reconstructed 3-D fluorescence images was performed using commercial software (AutoQuant X3, Media Cybernetics, Rockville, MD, USA).

### Incorporation of ethynyl uridine (EU)

Ethynyl uridine (EU) was incorporated using a Click-iT™ RNA Alexa Fluor™ 594 Imaging Kit (Thermo Fisher Scientific Inc., Waltham, MA, USA). Cells transfected with the expression vectors nAC-GFP, nLifeact-GFP, or GFP (control) were treated with 100 μM VP16 for 16 h and subsequently incubated with 1 mM EU for 1 h. The cells were fixed with 4% PFA and permeabilized with 0.1% Triton X-100 in PBS. After washing with PBS, the cells were incubated with a Click-iT reaction cocktail containing Alexa Fluor 594 azide for 30 min and then washed with the Click-iT reaction rinse buffer.

### Statistical analysis

Statistical comparisons were performed using Welch’s two-sided t-test. *p* < 0.05 was considered to be a significant difference.

## Results

### Depletion of p53 promotes the formation of nuclear actin filaments upon treatment with DNA-damaging agents

**Fig. 1:**
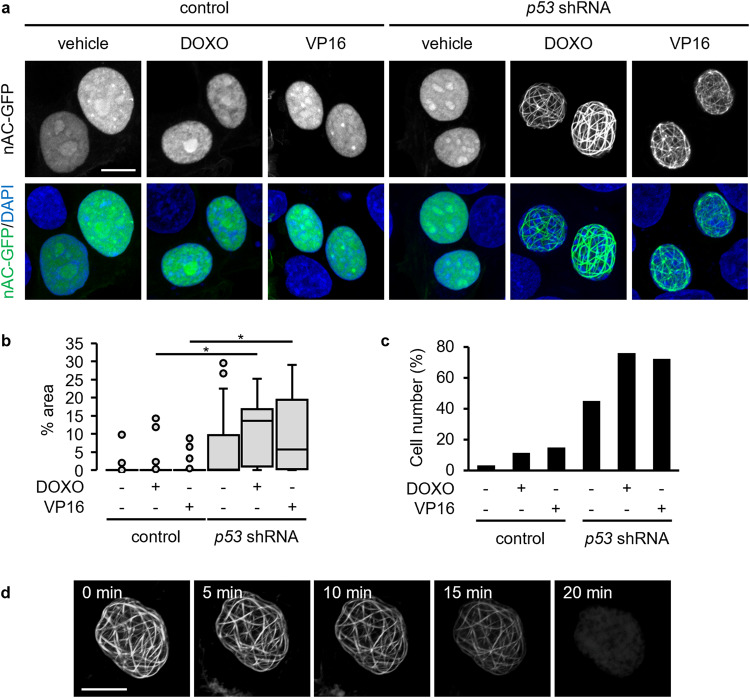
Knockdown of p53 promotes nuclear actin filament formation in nAC-GFP-expressing MCF-7 cells by treatment with DNA-damaging agents. MCF-7 cells expressing control (**a**–**c**) or *p53* shRNA (**a**–**d**) were transfected with nAC-GFP expression vector and subsequently treated with or without DOXO (1 μg/mL) or VP16 (100 μM) for 16 h. **a** Confocal images of nAC-GFP (gray/green) and DNA stained using DAPI (blue). The Z stack projections of 30 central plane images acquired at 0.1 μm intervals were obtained. The scale bar is 10 μm. **b**, **c** For each treatment, the nuclear area occupied by actin filaments was measured. The horizontal line represents the median, and the upper and lower whiskers represent the maximum and minimum values, respectively (**b**). The cells with non-uniform nAC-GFP localization in ≥1% of their area were classified as nuclear actin filament-containing cells, whereas those with non-uniform nAC-GFP localization in <1% were classified as nuclear actin filament-free cells (**c**). *N* ≥ 60 (**b**, **c**) for each treatment. Asterisks, *p* < 0.005. **d** The cells treated with DOXO for 16 h were incubated in the presence of mycalolide B (1 μM) for 20 min. The disappearance of the nuclear actin filament was observed using a time-lapse confocal microscope for 20 min.

**Fig. 2:**
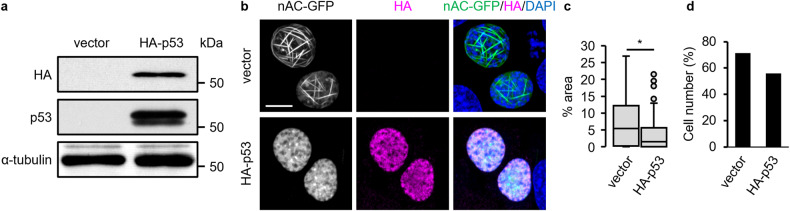
Reintroduction of p53 suppressed the DOXO-induced formation of nuclear actin filaments in p53 knockdown cells. MCF-7 cells expressing *p53* shRNA were cotransfected with nAC-GFP expression vector and HA-p53 expression vector and subsequently treated with DOXO (1 μg/mL) for 16 h. **a** Cell lysates were subjected to immunoblot analysis with antibodies against HA, p53, and α-tubulin as a loading control. **b** Confocal images of nAC-GFP (gray/green), HA-p53 stained using HA antibody (magenta), and DNA stained using DAPI (blue) are shown. The Z stack projections of 30 central plane images acquired at 0.1 μm intervals were obtained. The scale bar is 10 μm. **c** For each treatment, the nuclear area occupied by actin filaments was measured. The horizontal line represents the median, and the upper and lower whiskers represent the maximum and minimum values, respectively (**d**). The cells with non-uniform nAC-GFP localization in ≥1% of their area were classified as nuclear actin filament-containing cells, whereas those with non-uniform nAC-GFP localization in <1% were classified as nuclear actin filament-free cells (**d**). *N* ≥ 150 for each treatment. Asterisks, *p* < 0.005.

Many actin-regulating proteins exist not only in the cytosol but also in the nucleus [7, 8]. Considering that p53 influences cell behaviors through the regulation of the actin cytoskeleton [34, 35], we hypothesized that p53 might modulate the structure of nuclear actin filaments upon DNA-damaging stimuli. To address this issue, we examined the effect of shRNA-mediated p53 knockdown on the formation of nuclear actin filaments in human breast cancer MCF-7 cells bearing wild-type (WT) *p53*. The expression levels of p53 in DOXO-treated cells were evaluated by western blotting, which confirmed the efficient knockdown of p53 (Fig. S1). nAC-GFP, a GFP-tagged anti-actin nanobody fused with NLS, was used to visualize nuclear actin. When control cells without p53 depletion were treated with DOXO, nuclear actin diffused throughout the nucleoplasm regardless of DOXO treatment (Fig. 1a). In contrast, p53 knockdown cells formed a network of prominent long nuclear actin filaments on treatment with DOXO. The results were quantified by measuring the nuclear area occupied by the nuclear actin filaments (Fig. S2a, b), which showed that both the nuclear area occupied by nuclear actin filaments and the ratio of cells with nuclear actin filaments were markedly increased by DOXO treatment under p53-depleted conditions (Fig. 1b, c). Nuclear actin filaments were also formed by treatment with other DNA-damaging agents, namely, VP16 and cisplatin (CPT), in p53 knockdown cells (Fig. 1a–c, Fig. S3a–c). The induction of DNA damage in response to DOXO, VP16, or CPT was confirmed by γH2AX staining, and the γH2AX levels were significantly higher in p53 knockdown cells than in control cells (Fig. S1, S3d), which was consistent with the genome instability caused by the loss of p53 [41]. These results suggest that DNA damage induces the formation of nuclear actin filaments, which is abrogated by p53 activation. To examine whether the nuclear actin filaments stained with nAC-GFP comprised polymerized actin, DOXO-treated p53 knockdown MCF-7 cells expressing nAC-GFP were incubated with mycalolide B, an actin-depolymerizing agent. Within 20 min, nAC-GFP-labeled filaments were not detected, indicating that they comprised polymerized actin (Fig. 1d, Video 1). When the shRNA-resistant form of p53 and nAC-GFP were co-expressed in p53 knockdown MCF-7 cells, the reintroduction of p53 suppressed the DOXO-induced formation of nuclear actin filaments (Fig. 2a–d), further indicating that p53 plays a critical role in inhibiting the formation of nuclear actin filaments induced by DNA-damaging stimuli. The effect of p53 on the nuclear actin filament formation in response to DOXO treatment was verified in A549 human lung cancer cells and primary mouse embryonic fibroblasts (MEFs). Consistent with the results of the p53 knockdown in MCF-7 cells, p53 knockdown in A549 cells (Fig. S4a) and p53 knockout in MEFs (Fig. S5a) markedly enhanced the DOXO-induced formation of nuclear actin filaments in these cells (Fig. S4b–d, S5b–d). Overall, these results reveal that p53 depletion promotes the formation of nuclear actin filaments upon DNA damage.

### Caspase-1 is involved in the p53-dependent suppression of nuclear actin filament formation

**Fig. 3:**
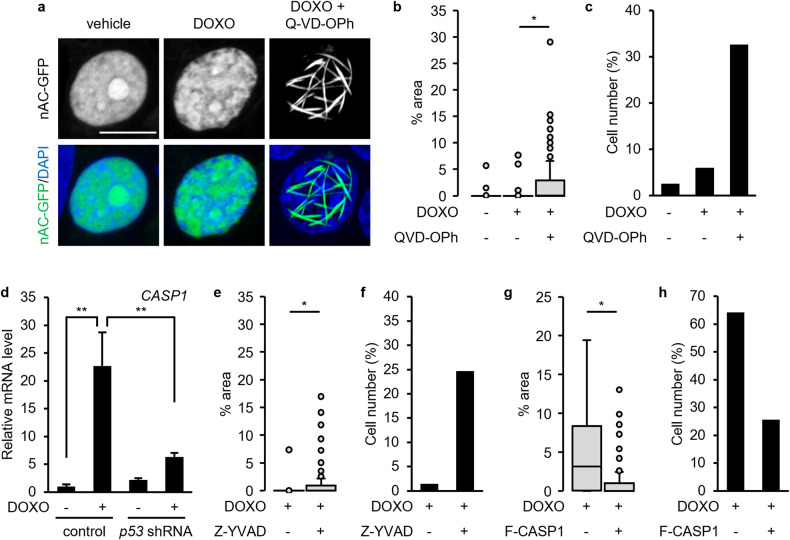
Caspase inhibitors promote the DOXO-induced formation of nuclear actin filaments in MCF-7 cells. **a**–**c**, **e**–**h** MCF-7 cells (**a**–**c**, **e**, **f**) or MCF-7 cells expressing *p53* shRNA (**g**, **h**) were transfected with nAC-GFP expression vector and subsequently treated with or without DOXO (1 μg/mL) and pan-caspase inhibitor Q-VD-OPh (100 μM) (**a**–**c**) or caspase-1 inhibitor Z-YVAD-FMK (100 μM) (**e**, **f**) for 16 h. **g**, **h** The cells were cotransfected with Flag-tagged caspase-1 (F-CASP1) expression vector with nAC-GFP expression vector. **a** Confocal images of nAC-GFP (gray/green) and DNA stained using DAPI (blue) are shown. The Z stack projections of 30 central plane images acquired at 0.1 μm intervals were obtained. The scale bar is 10 μm. **b**, **c**, **e**–**h** For each treatment, the nuclear area occupied by actin filaments was measured. The horizontal line represents the median, and the upper and lower whiskers represent the maximum and minimum values, respectively (**b**, **e**, **g**). The cells with non-uniform nAC-GFP localization in ≥1% of their area were classified as nuclear actin filament-containing cells, whereas those with non-uniform nAC-GFP localization in <1% were classified as nuclear actin filament-free cells (**c**, **f**, **h**). *N* ≥ 89 (**b**, **c**), *N* ≥ 75 (**e**, **f**), and *N* ≥ 80 (**g**, **h**) for each treatment. **d** The expression of *CASP1*-encoding caspase-1 in the MCF-7 cells expressing control and *p53* shRNA was evaluated by quantitative real-time PCR. Each bar represents the mean ± S.D.; *n* = 3. Asterisks, *p* < 0.005; double asterisks, *p* < 0.05.

Subsequently, we investigated the mechanisms underlying nuclear actin filament formation in p53-depleted cells. p53 activates several caspases. Q-VD-OPh, a pan-caspase inhibitor, was used to examine whether caspase activity influenced the formation of nuclear actin filaments. Compared with the treatment with DOXO alone, co-treatment with DOXO and Q-VD-OPh increased the formation of nuclear actin filament in MCF-7 cells without p53 depletion (Fig. 3a–c), indicating that caspase activity is involved in the p53-dependent suppression of nuclear actin filament formation. Q-VD-OPh inhibited the activation of caspase-1, caspase-3, caspase-7, caspase-8, caspase-9, caspase-10, and caspase-12 [42, 43]. It has been reported that *CASP1* encoding caspase-1 is a direct target of p53 and that DOXO induces caspase-1 activation through p53 in MCF-7 cells [44], which lack caspase-3 expression [45]. Whilst caspase-1 is known to cleave rho-associated protein kinase 1 (ROCK1) [46], DOXO treatment induced ROCK1 cleavage, which was diminished by p53 knockdown or co-treatment with either Q-VD-OPh or Z-YVAD-FMK (Fig. S6a–c). This indicated that caspase-1 was activated in DOXO-treated MCF-7 cells in a p53-dependent manner. Furthermore, as reported previously [44], *CASP1* expression was increased upon the DOXO treatment, which was diminished by p53 knockdown (Fig. 3d, S4e). Treatment with the caspase-1 inhibitor Z-YVAD-FMK enabled the formation of nuclear actin filaments in p53-expressing MCF-7 cells in response to DOXO treatment (Fig. 3e, f, S6d). In contrast, ectopic expression of Flag-tagged caspase-1 attenuated the DOXO-induced formation of nuclear actin filaments in p53 knockdown cells (Fig. 3g, h, S6e, f). These results demonstrated that caspase-1 acts downstream of p53 to suppress nuclear actin filament formation. Notably, Q-VD-OPh treatment induced the formation of nuclear actin filaments to a greater extent than Z-YVAD-FMK treatment in DOXO-treated MCF-7 cells, suggesting that caspase-1 and other caspases are involved in the p53-dependent suppression of nuclear actin filament formation.

**Fig. 4:**
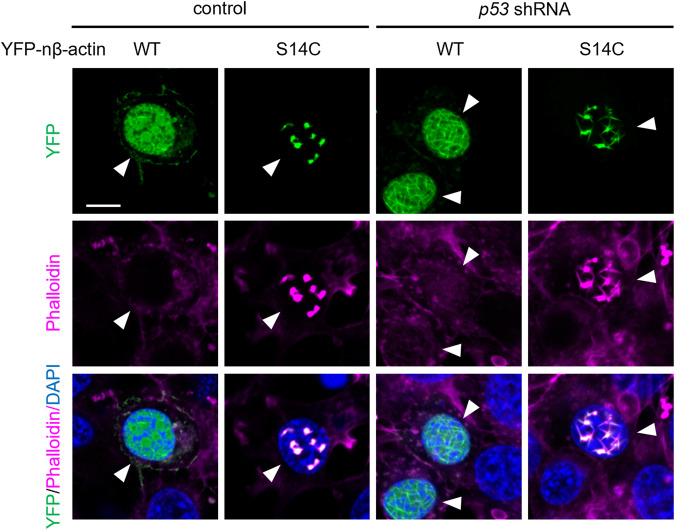
Depletion of p53 induces nuclear actin filament formation in YFP-nβ-actin-expressing cells upon treatment with DOXO. MCF-7 cells expressing control or *p53* shRNA were treated with or without DOXO (1 μg/mL) for 16 h. The cells were transfected with YFP-nβ-actin WT or S14C mutant expression vectors before treatment with DOXO. Confocal images of YFP-nβ-actin WT or S14C (green), F-actin stained using phalloidin (magenta), and DNA stained using DAPI (blue) are shown. The scale bar is 10 μm. Arrowheads show the transfected cells. Arrows indicate non-transfected cells.

Subsequently, we investigated whether an increase in the G-actin pool contributes to the promotion of nuclear actin filament formation. Accordingly, WT β-actin or a polymerization-favoring mutant of β-actin S14C, fused with NLS and tagged with YFP (YFP-nβ-actin WT or S14C), was expressed in MCF-7 cells. Similar to nAC-GFP, YFP-nβ-actin WT was uniformly distributed inside the nuclei of DOXO-treated cells without p53 depletion (Fig. 4). On the other hand, expression of YFP-nβ-actin S14C induced the formation of foci and not filaments in the nucleus, regardless of DOXO treatment (Fig. 4, S5). In p53 knockdown cells treated with DOXO, long nuclear filaments containing YFP-nβ-actin WT were observed (Fig. 4). In contrast, p53 knockdown cells expressing YFP-nβ-actin S14C showed formation of nuclear foci (Fig. S5), while DOXO treatment resulted in the formation of short and thin nuclear filaments emanating from the foci (Fig. 4). These results indicate that an increase in the nuclear actin pool was insufficient to induce nuclear actin filament formation. Conversely, both a reduction in p53 levels and DOXO treatment are required for the formation of nuclear actin filaments. Notably, while the foci and short filaments formed in YFP-nβ-actin S14C-expressing cells were strongly stained with phalloidin, the YFP-nβ-actin WT-containing filaments formed in p53 knockdown cells were hardly recognized by phalloidin. This implied that the molecular structures of F-actin differ between nuclear actin filaments and nuclear actin foci.

### Expression of nLifeact-GFP induces structural changes of chromatin in p53-depleted cells under conditions of DNA damage

**Fig. 5:**
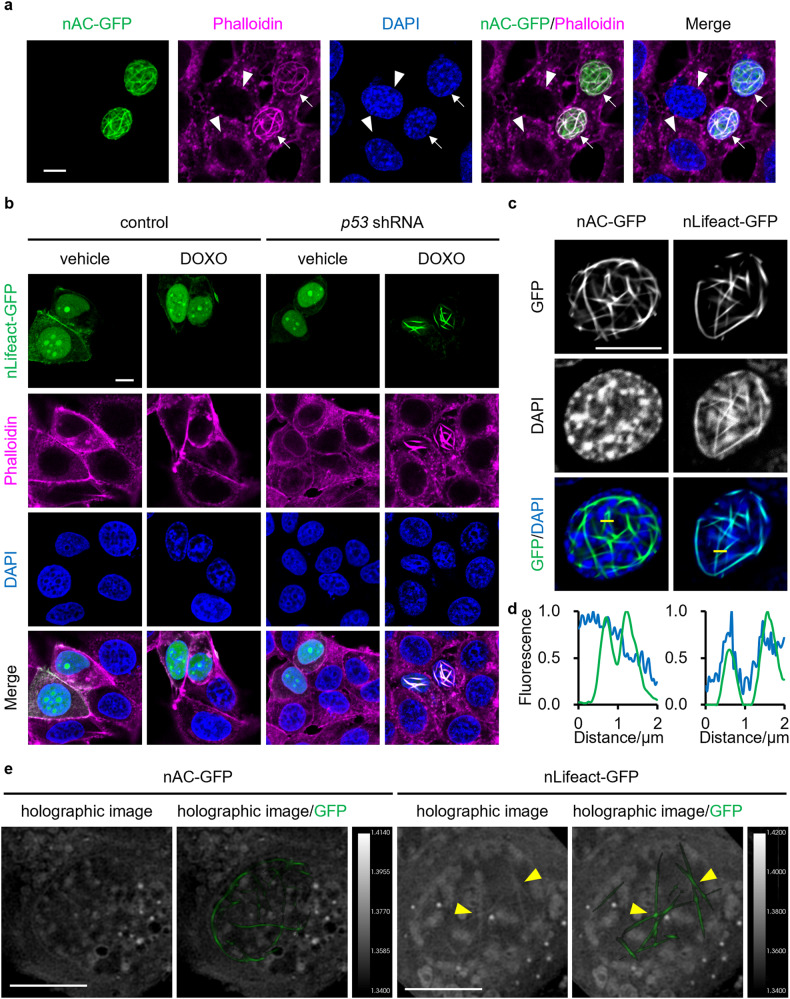
Expression of nLifeact-GFP induces structural changes of chromatin in DOXO-treated p53 knockdown MCF-7 cells. MCF-7 cells expressing control (**b**) or *p53* shRNA (**a**–**e**) were transfected with nAC-GFP (**a**, **c**, **e**) or nLifeact-GFP (**b**–**e**) expression vectors and subsequently treated with or without DOXO (1 μg/mL) for 16 h. **a**–**c** Confocal images of nAC-GFP or nLifeact-GFP (gray/green), F-actin stained using phalloidin (magenta), and DNA stained using DAPI (blue) are shown. **a**–**c**, **e** The scale bars are 10 μm. **a** The arrows show the transfected cells. The arrowheads show the non-transfected cells. **d** Line plots of nAC-GFP or nLifeact-GFP and DAPI fluorescence intensity (denoted by yellow lines) in (**c**). The intensity values were normalized to the maximum value of each fluorescence. **e** Images of 2D hologram (gray) and fluorescence visualized nuclear actin with nLifeact-GFP or nAC-GFP (green). The arrowhead shows the position of the nuclear actin bundle from nLifeact-GFP.

Under the background of p53 knockdown and DOXO treatment, the nuclear filaments formed in YFP-nβ-actin WT-expressing cells seemed to be thinner than those formed in nAC-GFP-expressing cells. Unlike YFP-nβ-actin WT-containing nuclear filaments, nuclear actin filaments labeled with nAC-GFP were clearly stained with phalloidin (Fig. 5a, arrow), whereas phalloidin-stainable filaments were not observed in cells that did not express nAC-GFP under the same p53-depleted and DOXO-treated conditions (Fig. 5a, arrowhead). Lifeact-GFP is widely used to visualize F-actin in living cells. Since it has been reported that Lifeact-GFP enhances actin polymerization [47], we examined whether NLS-fused Lifeact-GFP (nLifeact-GFP) induces the formation of phalloidin-stainable actin filaments in the nucleus. Similar to nAC-GFP-expressing cells, cells expressing nLifeact-GFP formed prominent nuclear actin filaments upon DOXO treatment in a p53 knockdown background, and nLifeact-GFP-labeled nuclear actin filaments were stained with phalloidin (Fig. 5b). Considering the previous paper which showed by electron microscopy that nLifeact expression induced the formation of thick bundles of nuclear actin filaments [48], our study implied that nAC and nLifeact promoted the bundling of actin filaments in the nuclei of DOXO-treated p53 knockdown cells. However, unlike the long and curved nAC-GFP-labeled nuclear actin filaments, the nuclear actin filaments labeled with nLifeact-GFP were relatively short and straight (Figs. 1a, d, 5a, b). Notably, we found that the expression of nLifeact-GFP modulated the chromatin structure based on staining with 4’,6-Diamidino-2-phenylindole dihydrochloride (DAPI; Fig. 5c, d). Some chromatin showed straight shapes that clearly overlapped with nLifeact-GFP-labeled nuclear actin filaments (Fig. 5c, e). The effect of nLifeact-GFP expression on the chromatin structure was further examined in living cells using holotomography, which can visualize macromolecular structures, such as chromatin and cell membranes but not actin filaments, by obtaining the refractive index (RI) distribution by passing low-energy light through the specimen with minimal perturbation [40, 49]. As shown in Fig. 5e, the holographic images showed that several straight structures were formed in the nucleus of nLifeact-GFP-expressing cells but not in the nAC-GFP-expressing ones that were depleted of p53 and treated with DOXO; these straight structures were colocalized with nLifeact-GFP fluorescence. As actin filaments cannot be detected by holographic microscopy, the straight structures observed in the holographic images likely represent chromosomal DNA associated with nLifeact-GFP-decorated nuclear actin filaments. Overall, these results suggest that nLifeact-GFP promotes the formation of nuclear actin filaments, thereby inducing structural changes in the chromatin of DNA-damaged and p53-depleted cells.

### Expression of nLifeact-GFP reduces the transcriptional activity in p53-depleted cells under the condition of DNA damage

**Fig. 6:**
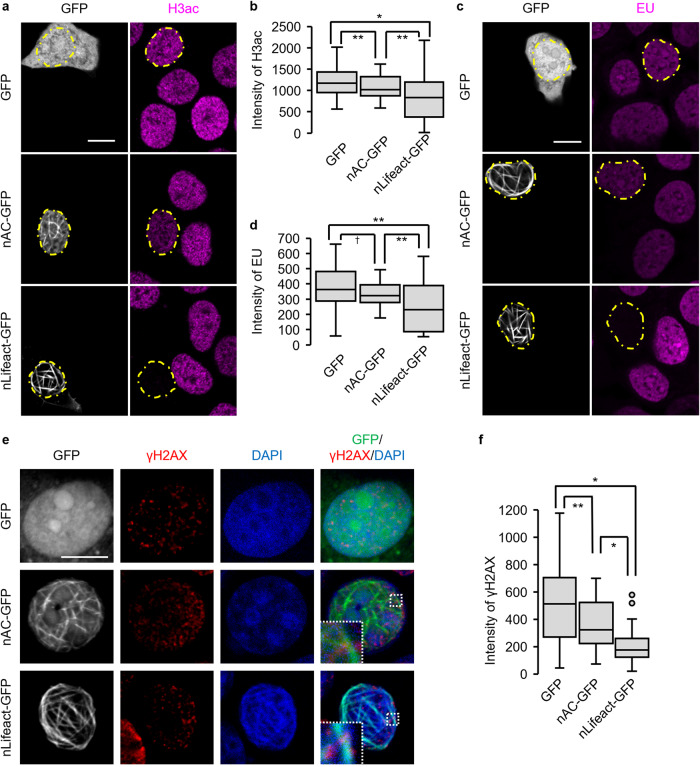
Expression of nLifeact-GFP suppresses RNA synthesis in p53 knockdown MCF-7 cells treated with DNA-damage agent. MCF-7 cells expressing p53 shRNA were transfected with GFP, nAC-GFP, or nLifeact-GFP expression vectors and subsequently treated with DOXO (1 μg/mL) (**a**, **b**) or VP16 (100 μM) (**c**–**e**) for 16 h. **c**, **d** The cells were incubated with 1 mM EU and then labeled with Alexa Fluor 594 azide by Click-iT reaction after fixation. **a**, **c** Confocal images of GFP, nAC-GFP, and nLifeact-GFP (gray), acetylated histone H3 at K9 (H3ac; magenta), and EU (magenta). The nuclei of the transfected cells are enclosed by a dotted line. The scale bar is 10 μm. **b**, **d** The intensity values of fluorescence of acetylated histone H3 or EU in the cells expressing GFP, nAC-GFP, or nLifeact-GFP. The horizontal line represents the median and the upper and lower whiskers represent the maximum and minimum values, respectively. *N* ≥ 36 and *N* ≥ 31 for each experiment. Each bar represents the mean ± S.D. Asterisks, *p* < 0.005; double asterisks, *p* < 0.05; dagger, *p* = 0.0594. **e** Confocal images of GFP, nAC-GFP or nLifeact-GFP (gray/green), γH2AX (red), or DAPI (blue). The scale bar is 10 μm. **f** The intensity values of fluorescence of γH2AX in the cells expressing GFP, nAC-GFP, or nLifeact-GFP. The horizontal line represents the median and the upper and lower whiskers represent the maximum and minimum values, respectively. *N* ≥ 24 for each experiment. Each bar represents the mean ± S.D. Asterisks, *p* < 0.01; double asterisks, *p* < 0.05.

The formation of phalloidin-stainable nuclear actin filaments often perturbs transcription by decreasing histone acetylation and RNA polymerase activity [15–17]. We examined the effect of nAC-GFP and nLifeact-GFP expression on the acetylation of histone H3 at K9, which correlates with transcriptional activation [50], and RNA synthesis. Expression of nAc-GFP or nLifeact-GFP, but not GFP alone, decreased the acetylation of histone H3 in the nucleus of p53 knockdown MCF-7 cells treated with DOXO (Fig. 6a). The level of acetylated histone H3 was significantly lower in the nLifeact-GFP-expressing cells than in the nAC-GFP-expressing cells (Fig. 6b). We evaluated RNA synthesis using the EU assay, wherein we used VP16 as a DNA-damaging agent to avoid the red autofluorescence of DOXO [51] which interfered with EU detection in our system. As was the case with DOXO treatment, VP16 treatment caused nLifeact-GFP-dependent modulation of the chromatin structure in p53-depleted MCF-7 cells (Fig. S8). The level of EU incorporation in nLifeact-GFP-expressing cells was significantly lower than that in cells expressing GFP alone or nAC-GFP, whilst nAC-GFP expression moderately reduced EU incorporation compared with GFP expression (Fig. 6c, d). Thus, in p53-depleted cells treated with DNA-damaging agents, nLifeact-GFP more strongly reduced the transcriptional activity than nAC-GFP, even though both probes induced the formation of phalloidin-stainable nuclear actin filaments. As nuclear actin filaments reportedly contribute to DNA repair under conditions of DNA damage stress [8], we examined the extent of DNA damage by immunostaining the DNA damage marker, γH2AX. Confocal microscopy showed that γH2AX foci localized on nAC-GFP-labeled nuclear actin filaments (Fig. 6e), which was consistent with a recent report by Cobb et al. [52]. In cells expressing nLifeact-GFP, chromatin exhibited straight structures that overlapped with nLifeact-GFP-labeled nuclear actin filaments, and γH2AX foci existed on both straight and other chromatins (Fig. 6e). The levels of γH2AX in cells expressing nAC-GFP or nLifeact-GFP were lower than those in cells expressing GFP alone. Notably, nLifeact-GFP-expressing cells showed a significantly lower intensity of γH2AX than nAC-GFP-expressing cells (Fig. 6f), suggesting that damaged DNA was repaired in cells forming nuclear actin filaments, with a higher extent in those expressing nLifeact-GFP. Thus, our findings suggest that the alteration of chromatin structure by nLifeact-induced bundles of nuclear actin filaments contributes to both transcriptional repression and DNA repair.

## Discussion

In this study, we used three actin probes, nAC-GFP, YFP-nβ-actin WT, and nLifeact-GFP, and showed that the loss of p53 promoted the formation of nuclear actin filaments upon DNA-damaging stimuli. In p53-depleted cells treated with the DNA-damaging agent, but not in control cells, YFP-nβ-actin WT exhibited filaments that formed network structures in the nucleus, which were hardly recognized by phalloidin (Fig. 4), while the filaments formed with nAC-GFP or nLifeact-GFP were clearly recognized with phalloidin (Fig. 5a, b). These results suggest that nAC-GFP and nLifeact-GFP modulate the structure of nuclear actin filaments at the molecular level. In contrast to YFP-nβ-actin WT and nAC-GFP, nLifeact-GFP caused a structural change in the chromatin to a straight shape along the nuclear actin filaments. Associated with this change, the acetylation of histone H3, RNA synthesis, and the γH2AX level were significantly reduced by the expression of nLifeact-GFP in p53-depleted DNA-damaged cells (Fig. 6). Lifeact interacts with the hydrophobic binding pocket of F-actin, wherein F-actin interacts with several actin-binding proteins, including myosin [53]. Actin filaments are stabilized in a larger extent by Lifeact than by AC [54]. These effects of Lifeact may highlight the differential influences of nLifeact and nAC on chromatin association with nuclear actin filaments as well as the inhibition of RNA synthesis and DNA repair. In aggressive cancer cells with a loss of p53 function, expression of nLifeact is expected to make cancer cells more resistant against anticancer drug-induced apoptosis because DNA damage is repaired more effectively in nLifeact-expressing cells. However, since the aberrant proliferation of cancer cells depends on the promotion of transcription, transcription attenuation by nLifeact-decorated nuclear actin filaments may lead to the inhibition of cancer cell proliferation. Even though the caspase-mediated apoptosis pathway, which is activated by DNA damage, is abrogated in p53-defective cancer cells [33], nLifeact would help prevent cancer progression by reducing cellular transcriptional activity. Thus, our findings potentially provide a novel strategy for enhancing the cytotoxicity of chemotherapy in aggressive cancer cells with a loss of p53 function.

Although we showed that the p53-dependent suppression of the formation of nuclear actin filaments was mediated by caspases, including caspase-1 (Fig. 3, S6d–f), the molecular mechanism underlying the promotion of nuclear actin filament formation through the loss of p53 remains unclear. Expression of YFP-nβ-actin WT did not induce the formation of filamentous nuclear actin structures in p53-expressing MCF-7 cells treated with the DNA-damaging agent (Fig. 4). Furthermore, when p53-expressing MCF-7 cells were treated with the DNA-damaging agent, the expression of the polymerization-favoring S14C mutant of YFP-nβ-actin did not induce the formation of fibrous structures of nuclear actin filaments, but induced that of nuclear actin foci instead. These results indicated that an increase in the amount of G-actin in the nucleus was insufficient to form nuclear actin filaments. In this study, we showed that the activation of caspases downstream of p53 is involved in the p53-dependent suppression of nuclear actin filament formation. As caspases are known to cleave several proteins that control actin polymerization [55], caspase-dependent deactivation or downregulation of actin regulatory proteins may be responsible for the p53-dependent suppression of nuclear actin filament formation in DNA-damaged cells. Further studies are required to test this possibility and elucidate the detailed molecular mechanism through which the loss of p53 promotes the formation of nuclear actin filaments.

### Supplementary information

Supplementary Information

Supplementary Video 1

Original Data File

Check list

## Data Availability

All data generated or analyzed during this study are available from the corresponding author on reasonable request.
