# Supplementary material for: Loss of p53 function promotes DNA damage-induced formation of nuclear actin filaments
Source: Cell Death Dis. 2023 Nov 25;14(11):766. doi: 10.1038/s41419-023-06310-0 (PMC10674001; doi:10.1038/s41419-023-06310-0)
Supplement: Supplementary file 1 — Supplementary Information [file 41419_2023_6310_MOESM1_ESM.docx]

**Takeru Torii et al.**

**Supplementary Information contains:**

**Supplementary Figures 1-8**

**
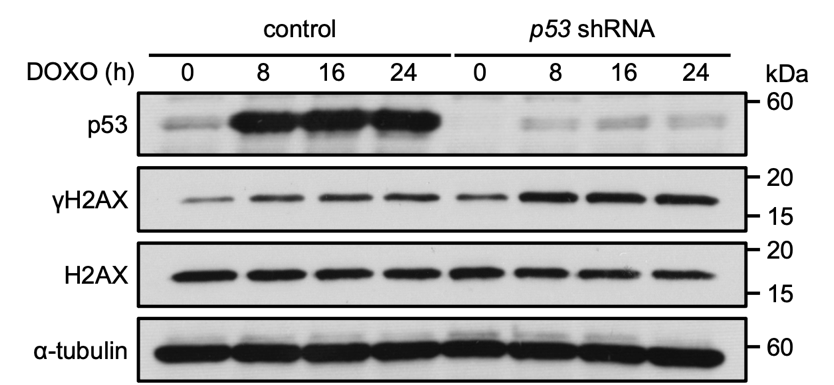
**

**Supplementary Figure S1. p53 expression is downregulated by infection of its shRNA expressing retroviruses in MCF-7 cells.**

MCF-7 cells expressing control or *p53* shRNA were treated with or without DOXO (1 μg/mL) for the indicated time periods. Cell lysates were subjected to immunoblot analysis with antibodies against p53, γH2AX, H2AX, and α-tubulin as a loading control.


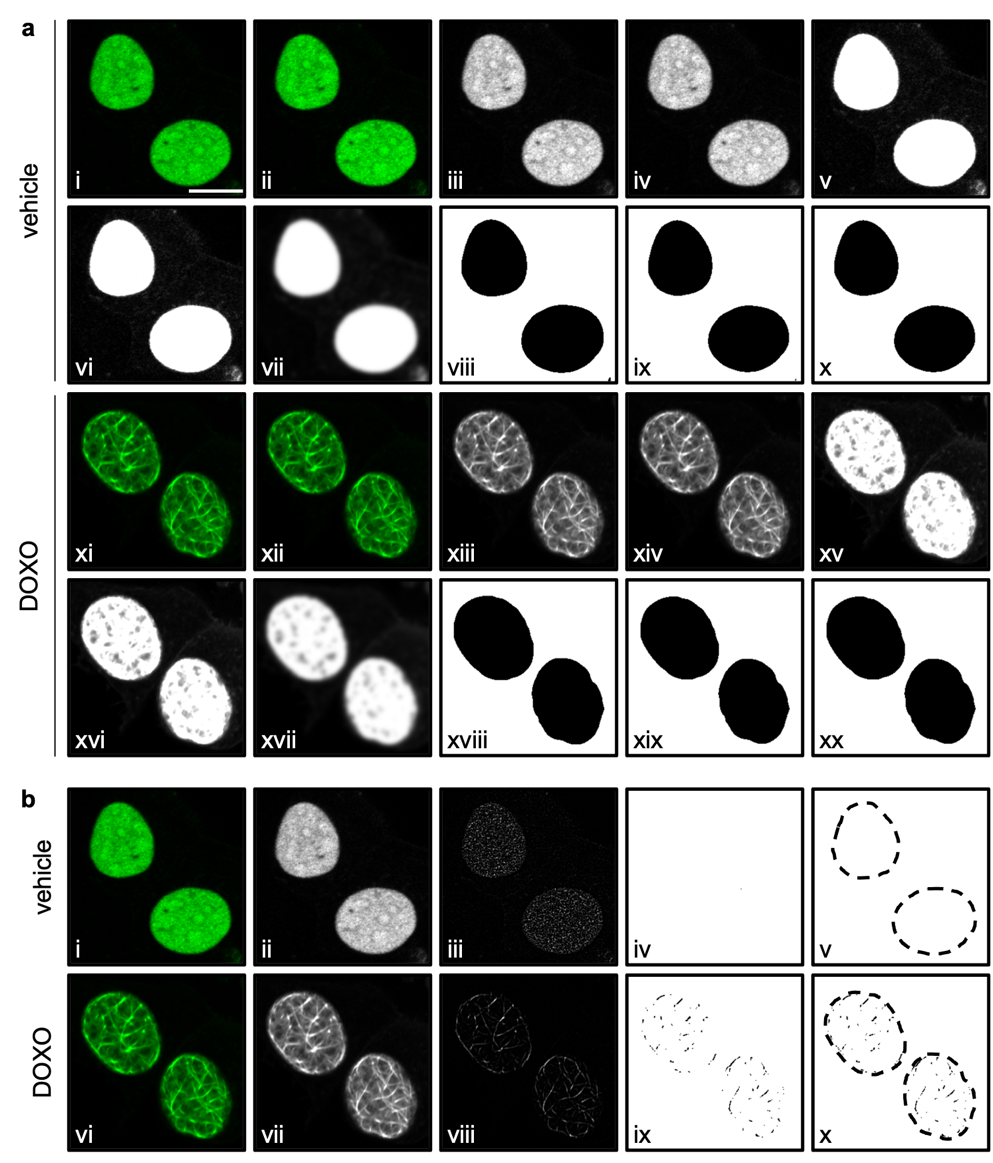


**Supplementary Figure S2. Identifying nuclear actin filaments in nuclear actin-stained cells.**

(**a–b**) MCF-7 cells expressing *p53* shRNA were transfected with the nAC-GFP expression vector and subsequently treated with (**a**xi-**a**xx, **b**vi-**b**x) or (**a**i-**a**x, **b**i-**b**v) without DOXO (1 μg/mL) for 16 h. (**a**) The method used to segment the nuclei in nuclear actin-stained cells. Using ImageJ software, segmentation of nAC-GFP was performed. Image analysis was performed as follows. The contrast of the signal was enhanced by increasing the number of saturated pixels by 0.35% (**a**ii, **a**xii), and the color of the original images changed from green (**a**ii, **a**xii) to gray (**a**iii, **a**xiii). The type of image was then changed from 16 to 8 bits (**a**iv, **a**xiv), and the signal was enhanced by multiplying it three times (**a**v, **a**xv). To remove noise and smoothen the signal, despeckle (**a**vi, **a**xvi) and Gaussian blur (**a**vii, **a**xvii) filters were sequentially applied. Subsequently, an automatic Otsu threshold was used to display the signal (**a**viii and **a**xviii), and the images were converted to binary images. The pixels located at the edges of the images were removed (**a**ix, **a**xix), and noise was eliminated using a despeckle filter (**a**x, **a**xx). For MCF-7 and A549 cells, the nuclear signal was determined as a signal that occupied an area greater than 60 μm^2^, respectively. For MEFs, the nuclear signal was determined as a signal that occupied an area greater than 80 μm^2^. Each signal was then registered as a region of interest (ROI) representing one nucleus. The scale bar is 10 μm. (**b**) The method used to determine the presence of nuclear actin filaments in cells. The presence of nuclear actin in the cells was detected using nAC-GFP, and the presence of nuclear actin filaments in the cells was determined as follows. Using ImageJ software, the color of the original images was first changed from green (**b**i, **b**vi) to gray (**b**ii, **b**vii); then, the background was subtracted using a rolling ball radius of 1 pixel (**b**iii, **b**viii). For these images, a threshold of 250 was used to detect the nuclear actin signal displayed as black over a white background (**b**iv, **b**ix). Using the predetermined ROI to identify each nAC-GFP-expressing cell (**b**v, **b**x), the presence of nuclear actin filaments was determined from the proportion of the nuclear area occupied by the nuclear actin signal. A nuclear area occupation of greater than 1% was used as the threshold to determine the presence of the nuclear actin filaments. The scale bar is 10 μm.

**
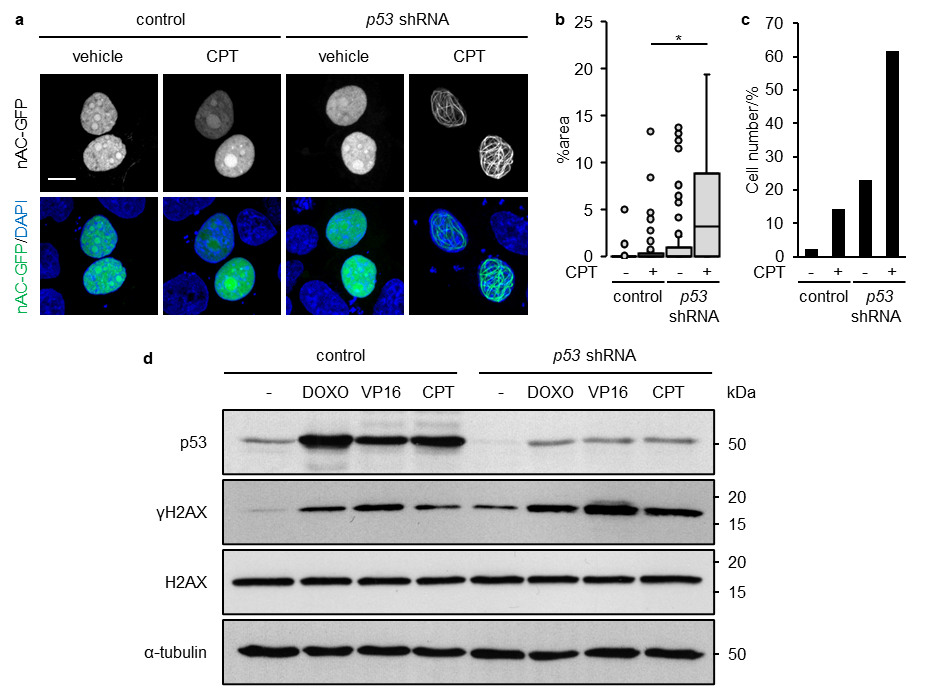
Supplementary Figure S3. Knockdown of p53 promotes the formation of nuclear actin filament in nAC-GFP-expressing MCF-7 cells by treatment with CPT.**

(**a–c**) MCF-7 cells expressing control or *p53* shRNA were transfected with the nAC-GFP expression vector and subsequently treated with or without CPT (100 μM) for 16 h. (**a**) Confocal images of nAC-GFP (gray/green) and DNA stained using DAPI (blue). Z-stack projections of 30 central plane images were acquired at 0.1-μm intervals. The scale bar represents 10 μm. (**b, c**) For each treatment, the nuclear area occupied by actin filaments was measured. The horizontal line represents the median, and the upper and lower whiskers represent the maximum and minimum values, respectively. (**b**) Cells with non-uniform nAC-GFP localisation in ≥ 1% of their area were classified as nuclear actin filament-containing cells, whereas those with non-uniform nAC-GFP localisation in < 1% were classified as nuclear actin filament-free cells. (**c**) N ≥ 55 for each treatment. Asterisks, *p* < 0.005. (**d**) MCF-7 cells expressing control or *p53* shRNA were treated with or without DOXO (1 μg/mL), VP16 (100 μM), or CPT (100 μM) for 16 h. Cell lysates were subjected to immunoblot analysis using antibodies against p53, γH2AX, H2AX, and α-tubulin as loading controls.


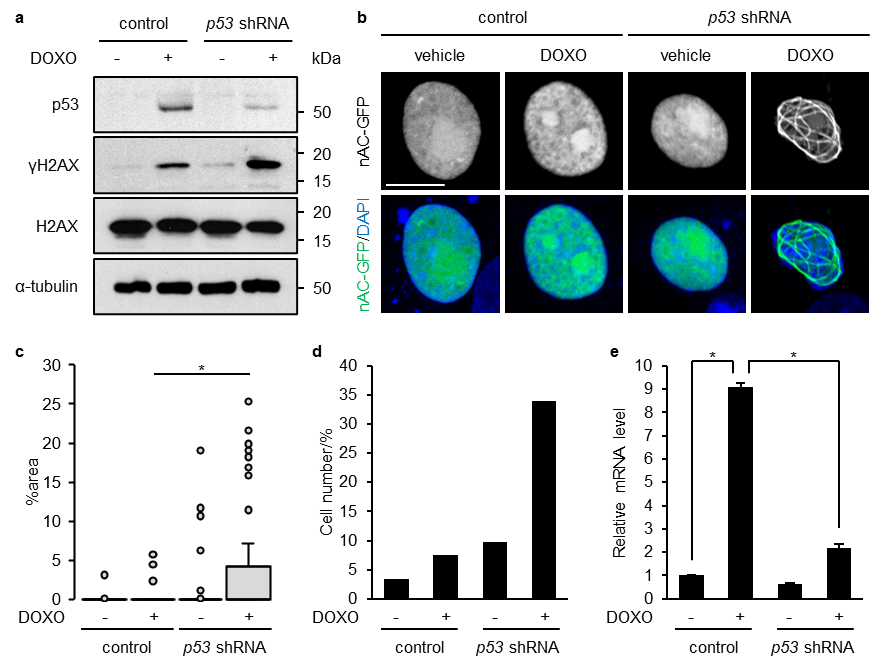


**Supplementary Figure S4. Knockdown of p53 promotes the formation of nuclear actin filament in nAC-GFP-expressing A549 cells by treatment with DOXO.**

(**a**) A549 cells expressing control or *p53* shRNA were treated with or without DOXO (1 μg/mL) for 16 h. Cell lysates were subjected to immunoblot analysis using antibodies against p53, γH2AX, H2AX, and α-tubulin as loading controls. (**b–d**) A549 cells expressing control or *p53* shRNA were transfected with the nAC-GFP expression vector and subsequently treated with or without DOXO (1 μg/mL) for 16 h. (**b**) Confocal images of nAC-GFP (gray/green) and DNA stained with DAPI (blue). Z-stack projections of 30 central plane images were acquired at 0.1-μm intervals. The scale bar is 10 μm. (**c, d**) For each treatment, the nuclear area occupied by the actin filaments was measured. The horizontal line represents the median, and the upper and lower whiskers represent the maximum and minimum values, respectively (**c**). Cells with non-uniform nAC-GFP localization in ≥ 1% of their area were classified as nuclear actin filament-containing cells, whereas those with non-uniform nAC-GFP localization in < 1% were classified as nuclear actin filament-free cells (**d**). N ≥ 53 for each treatment. (**e**) A549 cells expressing control or *p53* shRNA were treated with or without DOXO (1 μg/mL) for 16 h. The expression of *CASP1*, which encodes caspase-1, was evaluated by quantitative real-time PCR. Each bar represents the mean ± S.D.; n = 3. Asterisks, *p* < 0.005.


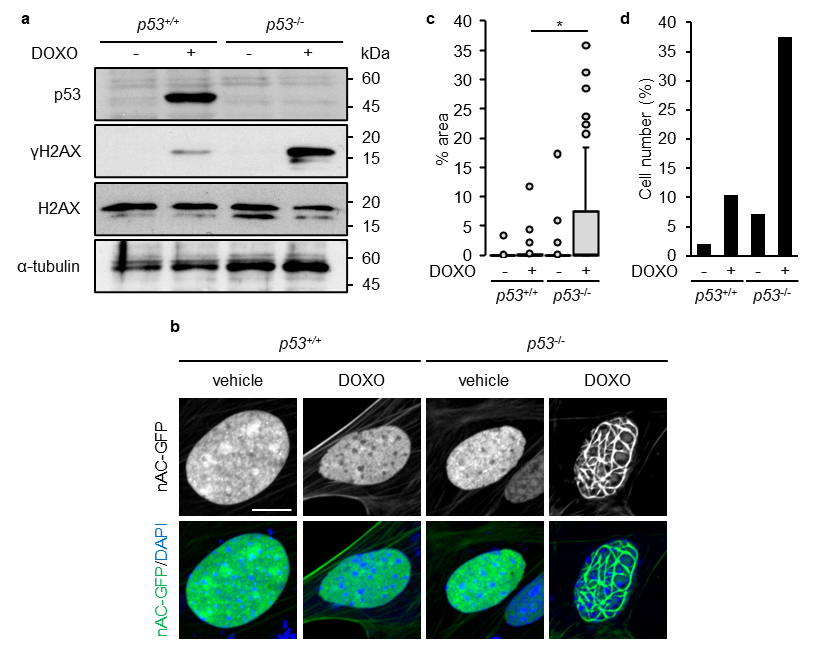


**Supplementary Figure S5. Formation of nuclear actin filaments is induced by treatment with DOXO in *p53*^−/−^ MEFs.**

(**a**) *p53*^+/+^ (WT) and *p53*^−/−^ MEFs were treated with or without DOXO (1 μg/mL) for 16 h. Cell lysates were subjected to immunoblot analysis with antibodies against p53, γH2AX, H2AX, and α-tubulin as loading controls. (**b–d**) *p53*^+/+^ (WT) and *p53*^−/−^ MEFs were transfected with the nAC-GFP expression vector and subsequently treated with or without DOXO (1 μg/mL) for 16 h. (**b**) Nuclear actin and DNA were visualized with nAC-GFP (green) and DAPI (blue), respectively. Z-stack projections of 30 central plane images acquired at 0.1 μm intervals were obtained. The scale bar is 10 μm. (**c, d**) For each treatment, the nuclear area occupied by actin filaments was measured. The horizontal line represents the median, and the upper and lower whiskers represent the maximum and minimum values, respectively (**c**). Cells with non-uniform nAC-GFP localization in ≥ 1% of their area were classified as nuclear actin filament-containing cells, whereas those with non-uniform nAC-GFP localization in < 1% were classified as nuclear actin filament-free cells (**d**). n ≥ 58 for each treatment. Asterisks, *p* < 0.005


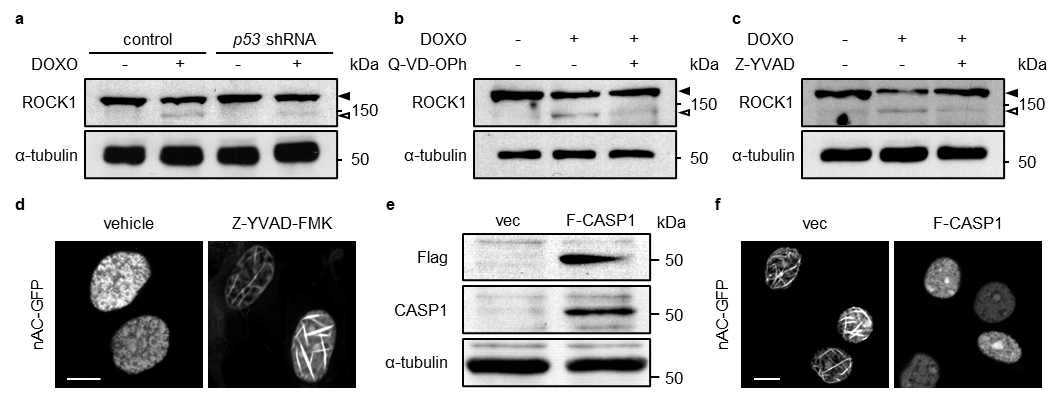


**Supplementary Figure S6. Caspase-1 suppresses the formation of nuclear actin filaments induced by DOXO treatment.**

(**a**) MCF-7 cells expressing control or *p53* shRNA were treated with or without DOXO (1 μg/mL). (**b, c**) MCF-7 cells were treated with or without DOXO (1 μg/mL), pan-caspase inhibitor Q-VD-OPh (100 μM) (**b**), and caspase-1 inhibitor Z-YVAD-FMK (100 μM) (**c**) for 16 h. (**a–c**) Cell lysates were subjected to immunoblot analysis with antibodies against ROCK1 and α-tubulin as loading controls. The black arrowhead indicates full-length ROCK1, whereas the white arrowhead indicates a cleaved fragment. (**d**) MCF-7 cells were transfected with the nAC-GFP expression vector and subsequently treated with DOXO (1 μg/mL) and Z-YVAD-FMK (100 μM) for 16 h. (**e**) MCF-7 cells expressing *p53* shRNA were transfected with Flag-tagged caspase-1 (F-CASP1) expression vector. (**e, f**) MCF-7 cells expressing *p53* shRNA were co-transfected with Flag-tagged caspase-1 (F-CASP1) and nAC-GFP expression vectors and subsequently treated with DOXO (1 μg/mL). (**d, f**) Confocal images of nAC-GFP. The scale bar is 10 μm. (**e**) Cell lysates were subjected to immunoblot analysis with antibodies against Flag, CASP1, and α-tubulin as loading controls.


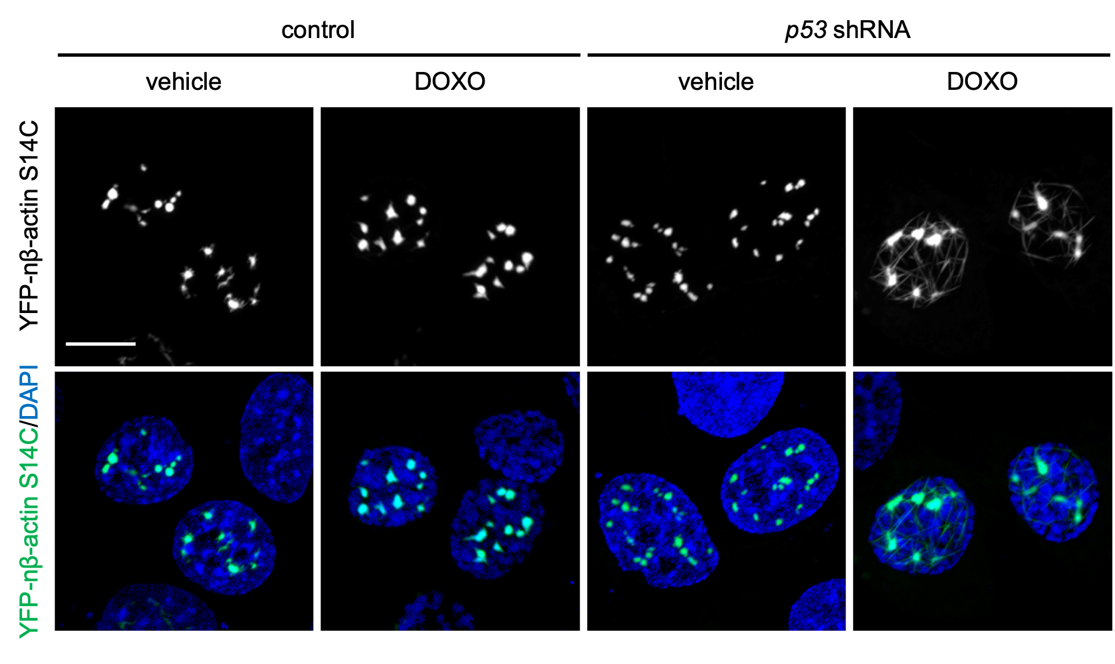


**Supplementary Figure S7. Formation of nuclear actin filaments with a fibrous structure was not observed in control MCF-7 cells expressing the YFP-nβ-actin S14C mutant.**

MCF-7 cells expressing control or *p53* shRNA were treated with or without DOXO (1 μg/mL) for 16 h. Cells were transfected with the YFP-nβ-actin S14C mutant expression vector before treatment with DOXO (1 μg/mL). Confocal images of YFP-nβ-actin S14C (gray/green) and DNA stained with DAPI (blue) are shown. Z-stack projections from confocal images of 30 central plane images acquired at 0.1 μm intervals were obtained. The scale bar is 10 μm.


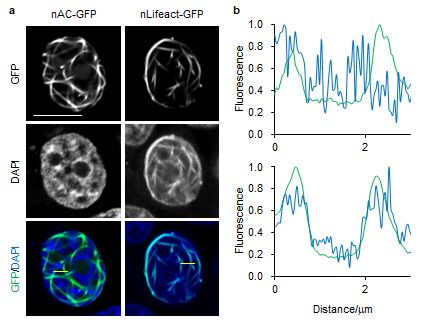


**Supplementary Figure S8. Expression of nLifeact-GFP modulates the chromatin structure based on staining with DAPI in VP16-treated MCF-7 cells expressing *p53* shRNA.**

MCF-7 cells expressing *p53* shRNA were transfected with nAC-GFP or nLifeact-GFP expression vectors and subsequently treated with VP16 (100 μM) for 16 h. (**a**) Confocal images of nAC-GFP or nLifeact-GFP (gray/green) and DNA stained with DAPI (gray/blue). The scale bar is 10 μm. (**b**) Line plots of nAC-GFP (upper panel) or nLifeact-GFP (lower panel) and DAPI fluorescence intensity (yellow lines) in (**a**). The intensity values were normalized to the maximum value of each fluorescence signal.
