# Supplementary material for: Loss of p53 function promotes DNA damage-induced formation of nuclear actin filaments
Source: Cell Death Dis. 2023 Nov 25;14(11):766. doi: 10.1038/s41419-023-06310-0 (PMC10674001; doi:10.1038/s41419-023-06310-0)

Fig. 2a

HA

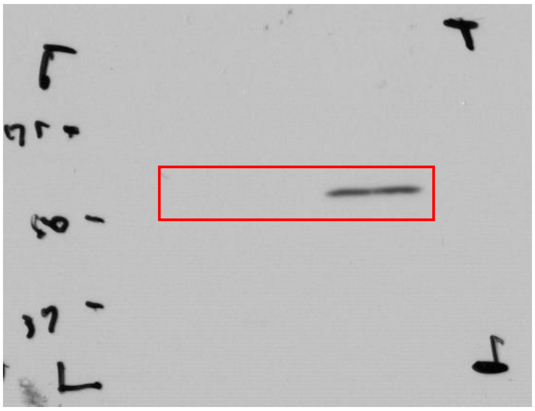

p53

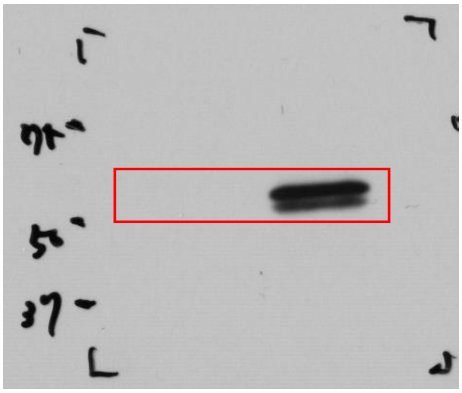

α-tubulin

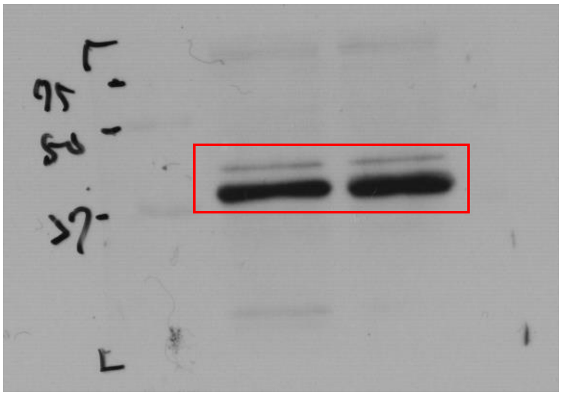

Fig. S1

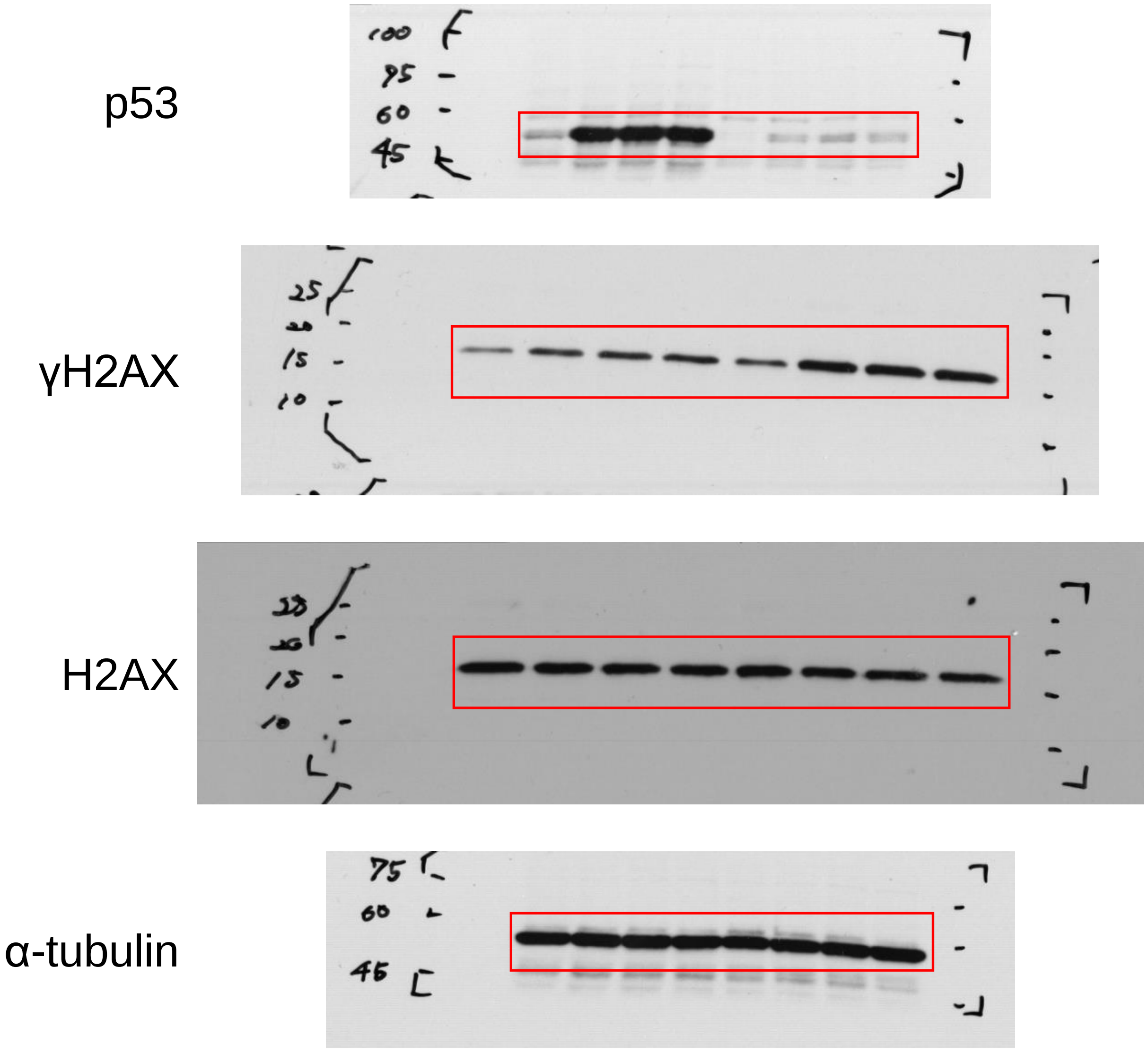

Fig. S3

p53

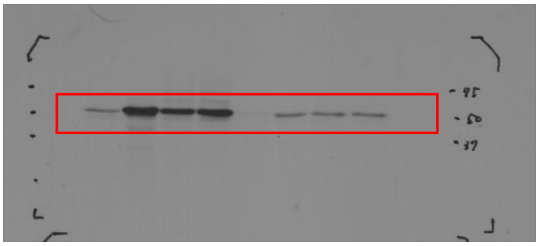

γH2AX

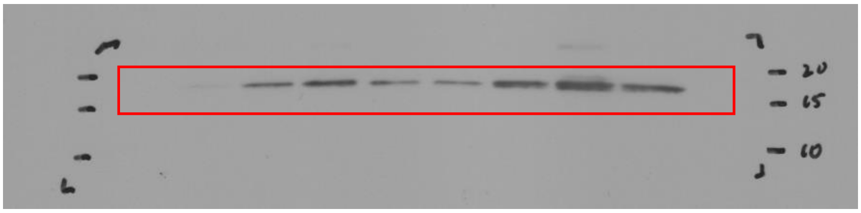

H2AX

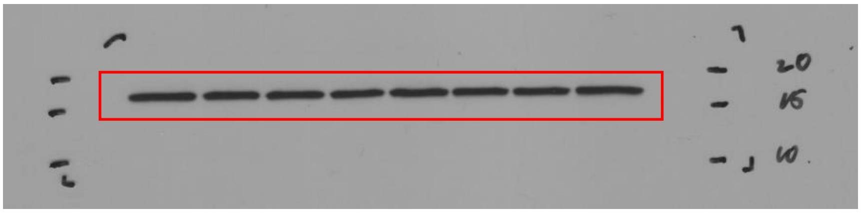

α-tubulin

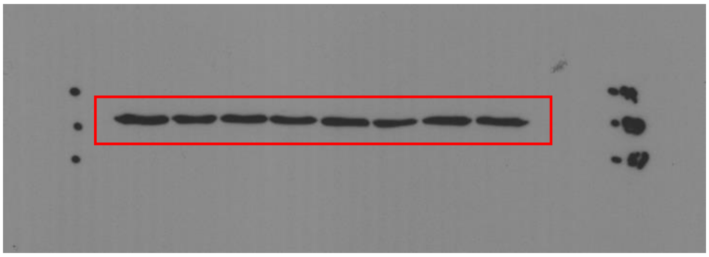

Fig. S4a

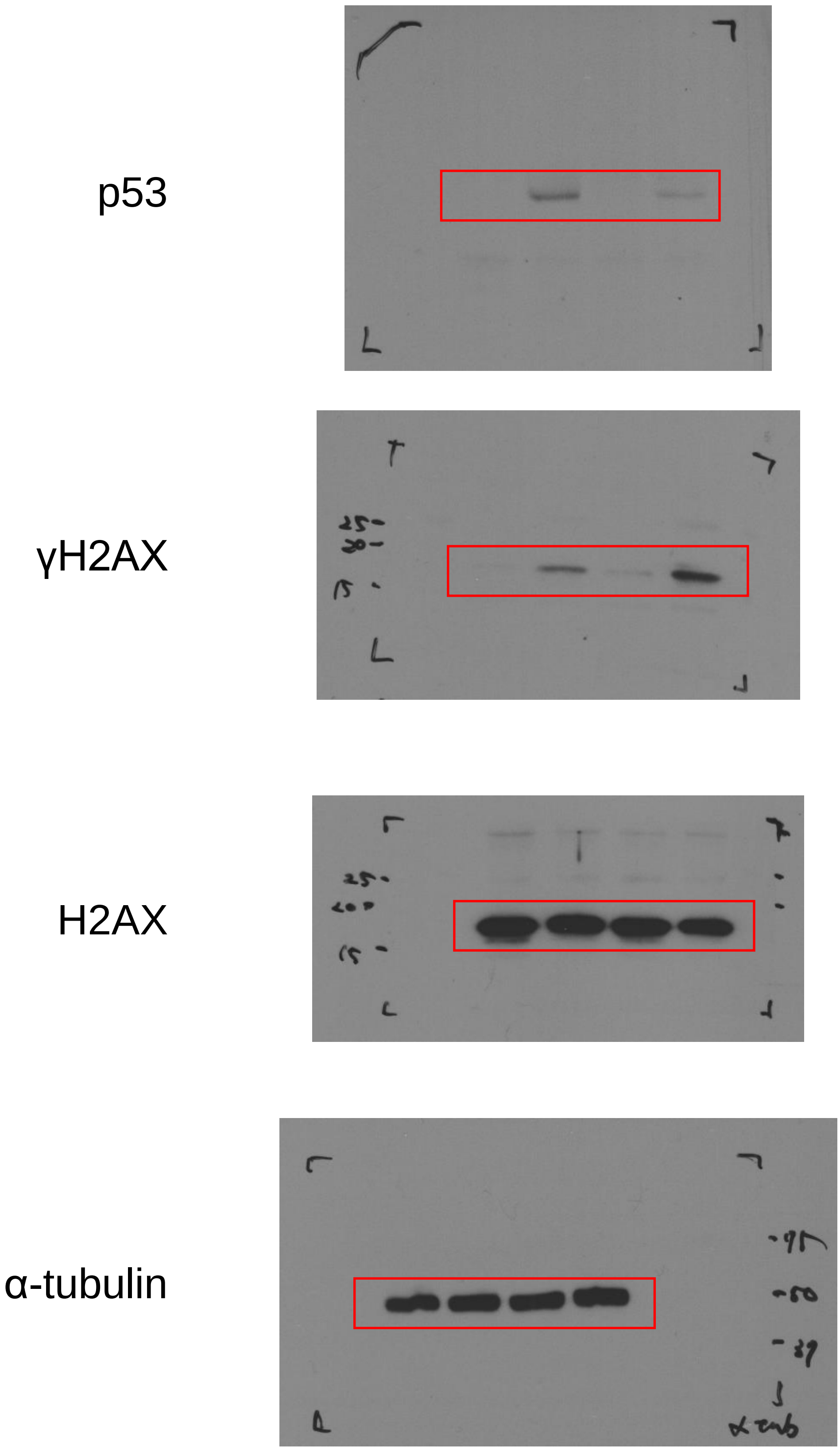

Fig. S5

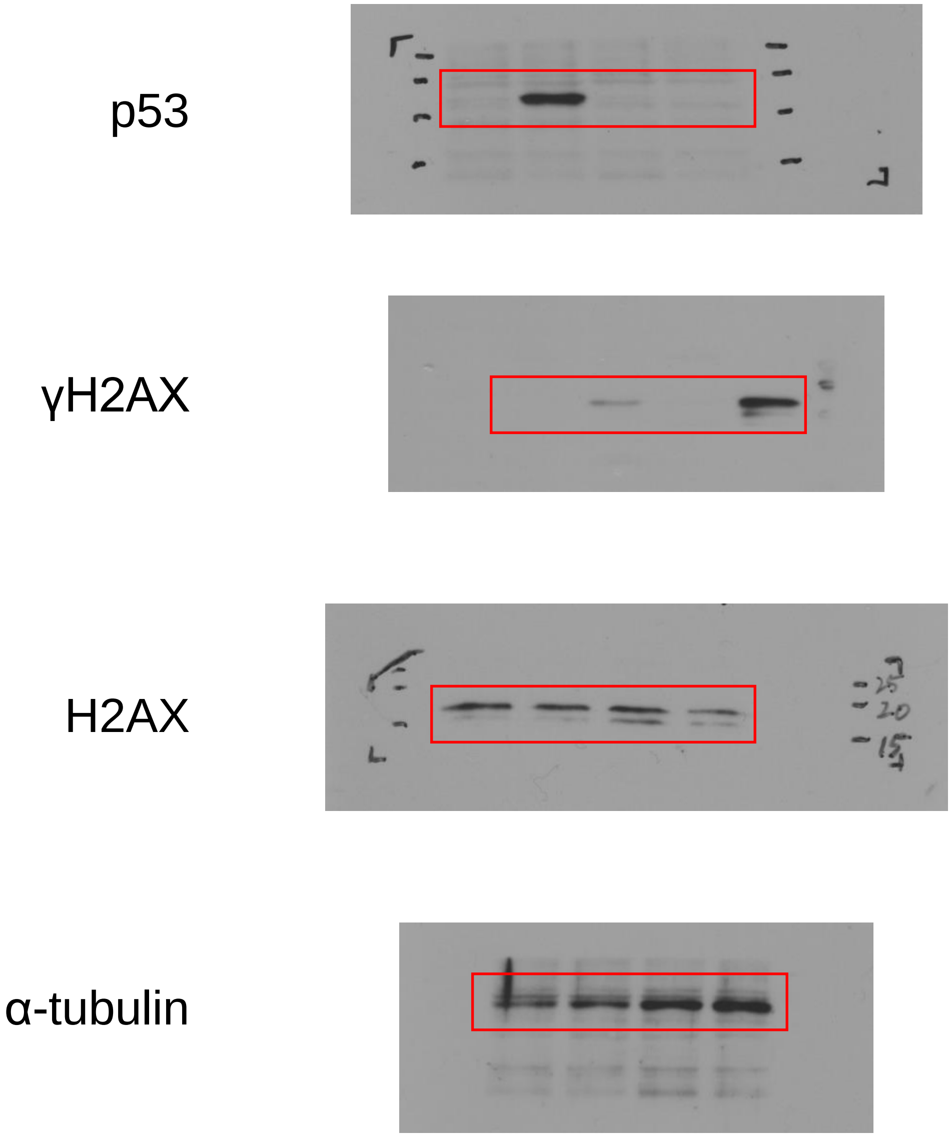

Fig. S6a

ROCK1

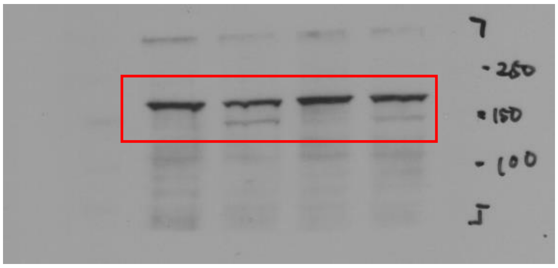

α-tubulin

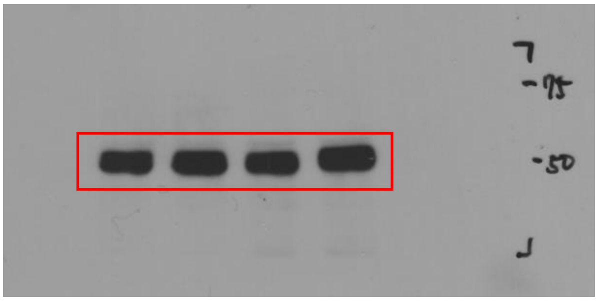

Fig. S6b

ROCK1

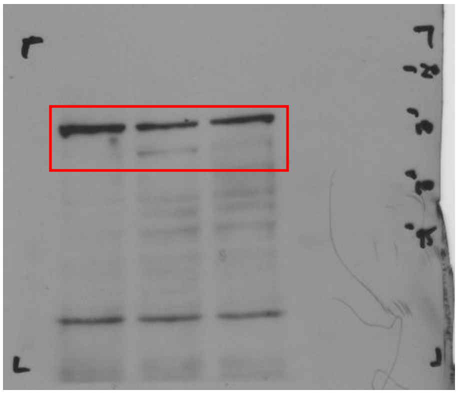

α-tubulin

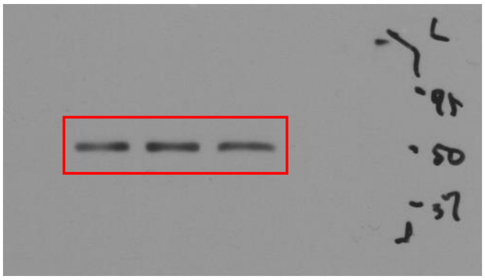

Fig. S6c

ROCK1

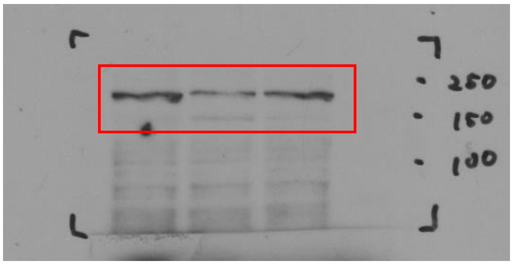

α-tubulin

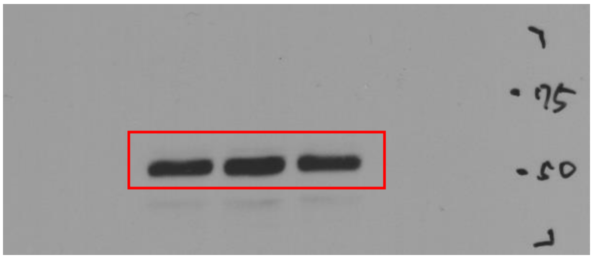

Fig. S6e

Flag

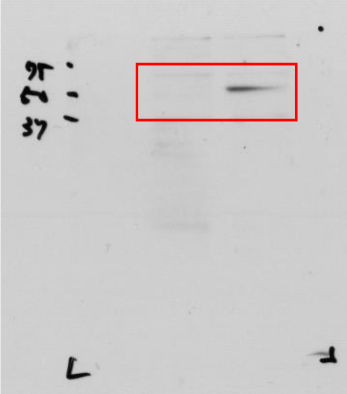

CASP1

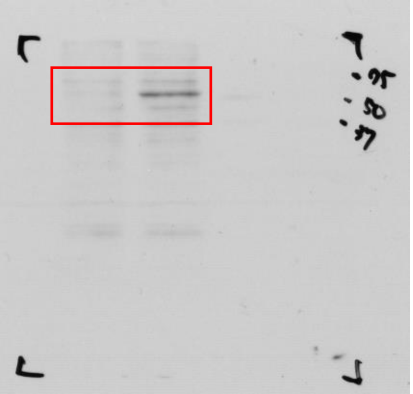

α-tubulin

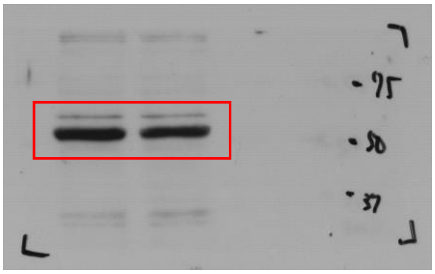

Supplement: Supplementary file 3 — Original Data File [file 41419_2023_6310_MOESM3_ESM.pdf]
